# Extra-pericardial tamponade following Wolf Mini-Maze procedure: a case report

**DOI:** 10.1186/s13019-015-0364-0

**Published:** 2015-11-05

**Authors:** James P. Bailey

**Affiliations:** Michigan State University College of Human Medicine, 965 Fee Rd. Room A-110, East Lansing, MI 48824 USA

**Keywords:** Atrial fibrillation, Cardiac tamponade, Warfarin, Hemorrhage, Anticoagulation

## Abstract

**Background:**

Extra-pericardial tamponade is a rare life threatening condition that has not previously been reported in association with Wolf Mini-Maze procedures. In this case, atypical presentation of cardiac tamponade caused by postoperative anticoagulation resulted in a second hospitalization, a second surgery, and delayed recovery time. The goal of this case report is to increase awareness about a life threatening complication that can occur following minimally invasive cardiac surgery.

**Case Presentation:**

A 60 year old male with long standing essential hypertension, who was recently treated for atrial fibrillation utilizing the Wolf Mini-Maze procedure, experienced a postoperative international normalized ratio increase from 3.6 to 5.3 over the course of six days. Fifteen days postoperatively, the patient experienced mild exercise intolerance, his condition rapidly progressed to a constellation of symptoms including severe exercise intolerance, dyspnea, hypotension, and near syncope. A diagnosis of cardiac tamponade was made, and the patient was re-admitted to the hospital. Attempts to reverse his warfarin anticoagulation with fresh frozen plasma and vitamin K were unsuccessful after 24 h. Video-assisted thoracotomy was performed to relieve the tamponade, and during surgery he was diagnosed with extra-pericardial tamponade caused by an extensive hematoma. Complications due to anticoagulation therapy required this re-admission, additional surgery, and delayed recovery. The patient has since recovered completely with no long term morbidities and is asymptomatic three years following initial presentation.

**Conclusions:**

This case marks the first time extra-pericardial tamponade has been reported post cardiothoracic intervention in English literature. Many surgical procedures require postoperative anticoagulation; in the past, warfarin has been the standard of care due to its purported reversibility. This case provides an example of the challenge presented when anticoagulating with warfarin, and the reversal of this anticoagulation.

## Background

Maze procedure subtypes (Cox-Maze III, Wolf Mini-Maze [MINI]) have been developed to treat and cure non-valvular atrial fibrillation (AF) [1–3] with a success rate of 96.6 % [2]. When successful, these procedures result in restoration of normal AV nodal transmission of atrial depolarization, thereby improving ventricular diastolic function and cardiac output. Cardiac rate is lowered when AF is converted to normal sinus rhythm [4]. If combined with left atrial appendage ablation, the nidus for thromboembolism is eliminated and the risk for stroke is reduced [1–3]. A secondary advantage to these procedures is the elimination of long term anticoagulation therapy for patients with AF and a resultant decrease in complications of major bleeding related to pharmacologic anticoagulation [1–7]. The MINI procedure is a less invasive approach to cure AF than the Cox-Maze III procedure. The MINI minimizes cosmetic changes, hospital stay duration, nosocomial infections, and reduces trauma [3]. Anticoagulant therapy (commonly warfarin [8]) is recommended for three months following any Maze procedure due to the risk of recurrent AF and the possibility of embolus [2]. Maze procedures are associated with postoperative atrial arrhythmias, fluid retention, pancreatitis, hemorrhage, pneumonia, and, in rare cases, cardiac tamponade [1, 2, 4]. Cardiac tamponade is compression of the heart, most commonly by fluid, preventing the filling of the right atrium and ventricle. The pericardial sac is the most common site for the development of cardiac tamponade. Cardiac tamponade can also occur from an external compression of the pericardial sac and the structures contained within it. This in turn inhibits adequate filling, decreases cardiac output, and results in inadequate perfusion. The adverse effects of cardiac tamponade include hypotension, syncope, severe exercise intolerance, pulseless electrical activity, cardiac arrest, and death. Cardiac tamponade has been reported in fewer than 1 % of maze procedures [2, 4]. Extra-pericardial cardiac tamponade has yet to be reported following any maze procedure.

A three month warfarin regimen is currently recommended following Maze procedures [2], however, anticoagulative therapy during the postoperative phase has been associated with hemorrhagic events [1], including major bleeding complications [5, 7, 8].

## Case presentation

### Significance/uniqueness of case

This case studies a patient who presented to the emergency department with near syncope caused by extra-pericardial cardiac tamponade sixteen days following a Wolf Mini-Maze procedure. To date there are no reports in English language medical literature, as accessed through PubMed, of extra-pericardial cardiac tamponade associated with any cardiothoracic surgical intervention. Extra-pericardial cardiac tamponade is a rare adverse event which has previously only been reported in association with blunt force trauma [9] and the repair of hiatal hernias [10]. There have been no reported cases of extra-pericardial cardiac tamponade due to hematoma formation following surgery.

### Patient description

The patient is a 60 year old Physician Assistant who is a male, a non-smoker, has mild alcohol intake, a long-standing history of essential hypertension, a BMI of 29.2, and a one month history of atrial fibrillation and atrial flutter. He had a catheter ablation for atrial flutter two months prior to his MINI procedure. He underwent the MINI procedure with no early postoperative complications. Immediately postoperatively, his hemoglobin was 14.1, hematocrit was 43.9, and INR (International normalized ratio) was 1.1 (Therapeutic range 2–3 [6–8]). His 24 h stay in ICU, followed by a 72 h stay in cardiac step down unit, was without incident and he was discharged on 5 mg Warfarin QD with instructions to return for regular INR monitoring. Upon discharge from the hospital, the patient was able to gradually resume his previous exercise regimen, including a daily two mile walk, with minimal loss of exercise tolerance. His INR was routinely monitored and was found to be 1.9 on the sixth postoperative day.

On the tenth postoperative day, his INR was found to be elevated at 3.6, so his dosage of warfarin was decreased to 2.5 mg QD. His diet had not changed prior to or following this change in INR. During the evening of the fifteenth postoperative day, the patient noted mild dyspnea upon exertion and he needed to slow down during his daily exercise. On the morning of sixteenth postoperative day, upon walking approximately 50 feet within his home the patient experienced near syncope. The patient’s wife, a retired pediatric Registered Nurse, was unable to auscultate a blood pressure with him in the standing or sitting position, but was able to palpate systolic blood pressure of 88 mmHg in the standing position. Upon auscultation of the patient’s chest, she detected a cardiac rub across the precordium accompanied by muffled heart sounds. His wife recommended ambulance transport but the patient refused, stating that he felt that the ambulance would be unable to find their rural home. His wife rapidly transported him in a private vehicle to the emergency department (ED) of a Level II trauma center.

### Intervention

Upon examination at the ED, the patient exhibited no signs of jugular venous distension or pulsus paradoxus, and an EKG performed showed normal sinus rhythm with no abnormalities. He was cognitively unimpaired. His hemoglobin was 12.8, hematocrit was 40.5, PTT was 49.8 (therapeutic range 25–35), and INR was 5.3 at 16:30. PA and lateral chest X-ray demonstrated blunting of the costophrenic angles in the lateral view and mild hypoventilatory changes, but showed no evidence of cardiomegaly, pulmonary congestion, or focal infiltration.

**Fig. 1:**
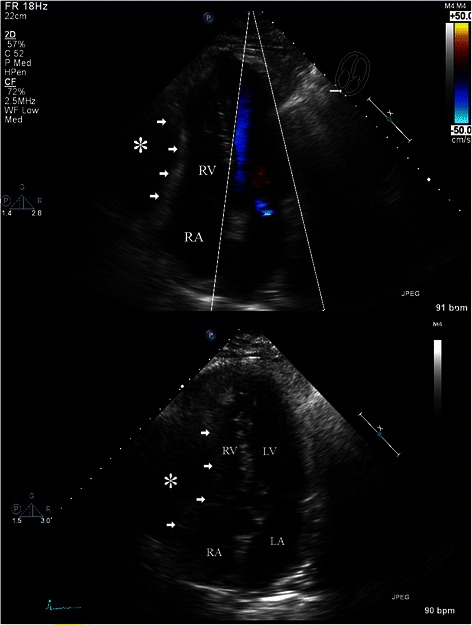
Apical Four Chamber Views Transthoracic Echocardiogram. These apical four chamber views on transthoracic echocardiogram demonstrate extensive extra-pericardial accumulation of blood and collapse of the right ventricle. Arrows: Compression of right ventricle, Asterisk: Hematoma, RA: Right atrium, RV: Right ventricle, LA: Left atrium, LV: Left ventricle

**Fig. 2:**
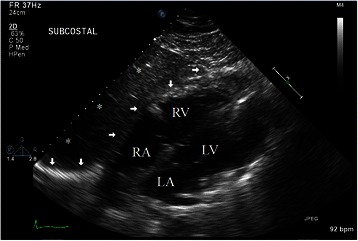
Subcostal view Transthoracic Echocardiogram. This subcostal view demonstrates the extra-pericardial accumulation of blood encompassing the majority of the anterior heart. Arrows: Borders of hematoma, Asterisks: Hematoma, RA: Right atrium, RV: Right ventricle, LA: Left atrium, LV: Left ventricle

Trans-thoracic echocardiogram demonstrated atypical motion of the interventricular septum, partial right ventricular collapse, and a large (6.2 cm) loculated effusion that layered from the inferior posterior wall, extended medially, and encompassing a portion of the anterior wall (Figs. 1 and 2). This effusion was thought to be within the pericardium. Ejection fraction was preserved at 55–60 %, and there were no significant valvular abnormalities. The ascending aorta was found to be minimally enlarged at 3.9 cm. There was no evidence of intracardiac shunt or cardiac masses. Preliminary diagnosis was early stage cardiac tamponade.

An unsuccessful attempt at reversal of his anticoagulation was made, utilizing two units of fresh frozen plasma and 10 mg IV vitamin K. Surgical consult with the original thoracic surgeon was obtained. The patient underwent video-assisted thoracotomy to relieve the tamponade the following afternoon, once anticoagulation had been reversed.

The thoracotomy was performed by reopening the original axillary incisions. Reopening of the old incisions revealed a “fair amount of old bloody fluid”, and an 800 cc posterior pleural effusion. The left lung was adhered to the posterior aspect of the pericardium. When the lung was separated from the pericardium, a loculated mass containing a pocket of blood located adjacent posteriorly to the pericardium was discovered, confirming the mass identified during the trans-esophageal echocardiogram. However, the mass was located extra-pericardially, contrary to the initial interpretation. The mass was an extensive hematoma which encompassed the posterior, medial, and a portion of the anterior pericardium.

Exploration within the pericardium revealed no additional blood. After ensuring no additional pockets of fluid remained, two Blake drains were inserted through separate stab incisions. Wounds were closed in layers with absorbable sutures and the patient was moved to the recovery room. No transfusions were necessary and no further complications ensued. Following initial recovery period, the patient was transferred to the cardiac stepdown unit.

### Response to treatment

Reversal of anticoagulation was attempted with two units of fresh frozen plasma and 10 mg IV vitamin K upon arrival to the emergency department. At 16:30 the patient’s INR remained elevated at 5.3, the following morning at 00:30 his INR was 2.2, at 08:30 his INR was 2.1, and at 14:45 his INR was 1.6. This demonstrated failure of reversal of anticoagulation [7]. His hemoglobin was 11.0 (down from 12.8), demonstrating significant blood loss.

The morning following his video-assisted thoracotomy, a portable chest x-ray demonstrated two well placed chest tubes on the left with no pneumothorax, “strandy” density in the left mid to lower lobes, discoid atelectasis in the right lung, and pleural fluid in the bases bilaterally. An outbreak of MRSA in the cardiac stepdown unit occurred during his hospitalization. Due to risk of infection, he was discharged two days following his thoracotomy with chest tubes in place and mini 500 Pleur-Evac to maintain chest tube function. He was anticoagulated with enoxaparin 60 mg subcutaneously BID for one week, pending follow up appointment. Four days following his thoracotomy, prior to chest tube removal, there was evidence of decreased lung volume, minimal atelectasis and scarring of the base of the left lung due to repeated chest tube insertion, and minimal bilateral pleural effusions causing blunting of the costophrenic angles.

Five days following his second operation the chest tubes were removed. Enoxaparin therapy was continued for three months during which he experienced no repeat bleeding. The patient experienced substantial exercise intolerance for several months following his second surgery.

Three years subsequent to both surgeries, the only persistent disability to the patient is bilateral paresthesia of the 5th thoracic dermatome. He has not developed additional bleeding events, and has resumed his prior activity level. He has not had recurrent episodes of atrial fibrillation or flutter.

## Discussion

Cardiac tamponade has rarely been reported in patients undergoing the MINI procedure concurrently with CABG; one patient has been reported in literature in multiple articles [4], and the complication does not appear to be directly attributable to the MINI procedure but rather ascending aortic rupture. As such, this case appears to be the first to report cardiac tamponade in absence of CABG with the MINI procedure. Current literature references cardiac tamponade with MINI procedures only once in a simulation study related to catheter ablation and Maze procedures, not an association of adverse effects reported following Maze procedures [11]. A search for extra-pericardial tamponade produced few results. One result describes a case of extra-pericardial tamponade during repair of a hiatal hernia that was not due to the formation of a hematoma [10]. Another result was that of a patient who experienced blunt force trauma resulting in hematoma formation in the mediastinum [9]. It is therefore clear that the presentation of cardiac tamponade following a MINI procedure is very rare, and that extra-pericardial tamponade due to hematoma formation caused by surgical intervention is extremely rare.

This case highlights the risks associated with postoperative warfarin anticoagulative therapy, including the difficulty controlling INR levels. Elevation of INR within the therapeutic anticoagulation range following reversal demonstrates that the frequently recited reversibility of warfarin has again been shown to be difficult and delayed without the addition of prothrombinex [7]. Multiple sources have indicated a statistically significant reduction in risk of thromboembolic events while at the same time a significant decrease in risk of major hemorrhagic events when anticoagulation therapy is based on a newer generacbtion anticoagulant [5, 8]. However, large clinical trials are still needed to demonstrate reversibility of these newer anticoagulants, and in absence of reliable reversibility, a bleed occurring while anticoagulated with one of these anticoagulants could be significantly worse.

## Conclusion

This case marks the first instance in English medical literature that extra-pericardial tamponade has been directly associated with cardiothoracic surgery. This case demonstrates the necessity for emergency department providers to maintain an index of suspicion of cardiac tamponade in patients who have undergone a Mini-Maze procedure and are still receiving anticoagulation therapy. Additionally, this case demonstrates the challenge, in both INR control and rapid reversal, which warfarin anticoagulation presents following Maze procedures [2].

### Consent

Written informed consent was obtained from the patient for publication of this Case report, patient history, laboratory information, and any accompanying images. A copy of the written consent is available for review by the Editor-in-Chief of this journal.

## Abbreviations

AF: Atrial Fibrillation
CABG: Coronary artery bypass graft surgery
ICU: Intensive care unit
INR: International normalized ratio
MINI: Wolf Mini-Maze procedure
